# Use of a Launderable Bed Barrier and Antibiotic Stewardship to Decrease Hospital Onset *Clostridioides difficile* Infections in an Acute Care Hospital: A Retrospective Pre-Post Case Study

**DOI:** 10.36469/001c.11149

**Published:** 2019-12-12

**Authors:** Edmond A. Hooker, Peter J. Mallow, Christine McKinney, Martin L. Gnoni, Francisco Fernandez Gonzales

**Affiliations:** 1 Xavier University, United States of America; 2 University of Cincinnati, United States of America; 3 Our Lady of Bellefonte, United States of America

**Keywords:** *Clostridioides difficile*, bacterial infection, bed barrier, antibiotic stewardship program

## Abstract

**Background:**

Hospital-onset *Clostridioides difficile* infection (HO-CDI) is a major source of morbidity and mortality. The objective of this research was to evaluate the reduction in HO-CDI through the use of a launderable bed barrier (BB) and an antibiotic stewardship program (ASP).

**Methods:**

A retrospective pre-post study was conducted at an acute care hospital in Kentucky. The preintervention period was September 2014 through March 2016. The BB and the ASP were introduced in April 2016, and the post-intervention period for this study ended September 2018. The rate of HO-CDI was calculated from the actual number of HO-CDI divided by the number of patient days each month. The number of defined daily doses of antibiotic therapy was measured each quarter. Hand disinfection compliance, length-of-stay (LOS), case mix index (CMI), and average age of patients were collected to control for confounding in the regression models.

**Results:**

There were 34 HO-CDIs and 42 672 patient days in the pre-intervention period and 31 HO-CDIs and 65 882 patient days in the post-intervention period. The average monthly count of HO-CDI was 1.79 (SD 1.51) and 1.03 (SD 0.96) during the pre- and post-periods, respectively. The average monthly rate (per 10 000 patient-days) was 7.94 (SD 6.30) in the pre-intervention period and 4.71 (SD 4.42) during the post-intervention period. The use of antibiotics decreased by 37% (*p* <0.0001) over the study period. The combination of the BB and the ASP were associated with a 59% (95% CI 36–96%, *p* 0.034) reduction in HO-CDI.

**Conclusions:**

The use of a launderable BB and the ASP were associated with a statistically and clinically significant reduction in HO-CDI in the acute care hospital setting.

## BACKGROUND

Despite attention from the healthcare system and governmental agencies, hospital-onset *Clostridioides difficile* infection (HO-CDI) has continued to be a major source of morbidity and mortality in both United States of America (USA) and internationally. It was estimated that in 2011 there were approximately 453 000 CDIs in the US, with an estimated 104 400 HO-CDIs and 29 300 deaths.^1^ A recent study of hospitals in the Emerging Infections Program showed that, while other healthcare acquired infections (HAIs) are decreasing, rates of HO-CDI were not significantly lower from 2011 to 2015.^2^ The National Healthcare Safety Network (NHSN) indicated that there has been a slight decrease in HO-CDI as of 2016; however, there were 95 530 HO-CDIs reported by 3605 acute care hospitals to NHSN for calendar year 2016.^3^ These HO-CDIs have added and estimate $4.8 billion in costs to acute care hospitals in the US.^4^ There has been a major emphasis on antibiotic stewardship programs (ASP) in order to decrease HO-CDI.^5^,^6^

Previous studies have documented that many surfaces (bed rails, bedside table, and phone) are still contaminated with bacteria after terminal cleaning, which is performed after the discharge of the patient.^7^–^9^ Hospital mattresses also remain contaminated after terminal cleaning.^10^–^13^ Additionally, there are a number of published studies indicating that many hospital mattresses are damaged and contain blood and bodily fluids.^14^,^15^ In 2017, after receiving over 700 reports of hospital mattress covers failing to prevent blood and body fluids from leaking into the mattress, the Food and Drug Administration (FDA) issued a guidance statement recommending routine inspections of all hospital mattresses.^16^ In 2018, the ECRI Institute identified bed and mattress contamination as one of their top ten healthcare hazards.^17^

Contaminated hospital mattresses have been linked to outbreaks and deaths.^18^ If the previous patient in a room was infected with CDI, the new patient was more than twice as likely to become infected with CDI.^19^ This risk was present even if the patient was simply colonized and asymptomatic.^20^,^21^ A previously published study demonstrated that use of a launderable bed-barrier (BB) in Long-term Acute Care Hospitals (LTACHs) was associated with a 50% reduction in CDIs.^22^ The objective of this study was to assess the effectiveness of a BB in conjunction with an ASP at an acute care hospital located in Kentucky, USA.

## MATERIALS AND METHODS

The study was set up as a retrospective pre-post study. The hospital for the current study was a 158-bed acute care hospital in Ashland, Kentucky, USA. The pre-intervention period was September 2014 through March 2016 and served as the baseline for establishing the rate of HO-CDI. The BB and an antibiotic stewardship program (ASP) were introduced in April 2016, and the post-period for this study ended September 2018. Approximately 3% of beds would not accommodate the bed barrier. However, all infections in the post-intervention period were counted, whether or not they occurred on a bed with a BB or not.

The HO-CDIs were identified according to the CDC’s National Healthcare Safety Network definitions. The HO-CDI was defined as a CDI infection starting on day 4 or later of hospital admission or within 4 weeks after discharge. Pressure ulcers were defined as 1) stage 1 pressure injury: non-blanchable erythema of intact skin 2) stage 2 pressure injury: partial-thickness skin loss with exposed dermis; 3) stage 3 pressure injury: full-thickness skin loss; 4) stage 4 pressure injury; full-thickness skin and tissue loss; 5) deep tissue pressure injury: (Unstageable, Stage 3 or Stage 4).^23^

The launderable BB (Soteria^®^) was manufactured by Trinity Guardion in Batesville, Indiana. The BB was manufactured using a polyurethane coated polyester, which is similar to the fabric used to manufacture mattress covers. The BB material was welded together and designed to fit a specific bed by manufacturer. Each different bed style requires its own style of BB, and the BB not only covers the mattress but also the bed deck (the metal surface upon which the mattress rests). The cover allows for full operation of each bed. The BB was removed after each patient discharge and laundered using a multistep process at the same commercial laundry utilized for all linens at the hospital. Each cover was laundered using a validated process that includes detergent, bleach, hot water (71°C), agitation, and multiple rinse cycles. The process has been shown to remove 99.9999% of bacteria and *C. difficile* spores from the cover.^24^ The cover was then dried using heat. Finally, after each cover was cleaned, it was inspected using a light table to identify and repair any damage. The cover was then reverse rolled and returned to the hospital. The hospital used Hill-Rom beds (VersaCare^®^, Total Care^®^, and Progressa^®^). The mattresses for these beds consisted of either a foam core or air cells/bladders and a polyurethane coated nylon cover, which was manually disinfected but not removed between patients. After initial training, there was not monitoring of the installation of the cover between patients.

The hospital contracted with the same environmental services (EVS) company during all periods of the study. Quaternary ammonia compounds were used for terminally cleaning of all rooms, except for isolation rooms. Isolations rooms were cleaned with hydrogen peroxide disinfecting solution (Oxycide^®^). There were no changes to terminal cleaning procedures during the study. Prior to May of 2018, the majority of the testing was by nucleic acid amplification tests (NAAT) done by polymerase chain reaction (PCR) (Biofire^®^) starting in March 2014 and Gastrointestinal Panel (Biofire^®^) starting in June 2016. Although the Infectious Disease Society of America recommends the use of NAAT alone or a multistep process with NAAT and testing for toxin, in May 2018, if *C. difficile* was suspected on day 4 of hospitalization or after, testing was done using ELISA for toxin A & B.^5^

The hospital initiated an ASP at the same time as the use of the BB. The ASP was informed by the Infectious Diseases Society of America (IDSA) and Society for Healthcare Epidemiology of America (SHEA) evidence-based guidelines.^25^ The ASP involved an education program for all physicians and physician assistants regarding the importance of *C. difficile* colonization and of not treating every positive urine culture. Handwashing compliance required washing in and out of a patient room to be counted. Trained observers performed more than 100 handwashing observations per month. Further, all antibiotic orders are reviewed by a pharmacist daily and the infectious disease physicians weekly. If antibiotics were deemed inappropriate, the treating physician was advised to either discontinue the antibiotics or request a consult from the infectious disease physicians. When fluoroquinolones were ordered and there was no good indication, the treating physician was made aware of the many risks of using fluoroquinolones, including CDI. Treating physicians were advised to utilize probiotics when antibiotics were prescribed, especially when using antibiotics known to have higher risk for CDI. CDI cases with associated diarrhea (CDAD) were treated with vancomycin, and this remained constant during all the study.

Patients with suspected cases of CDI were immediately placed in single room, pending test results. Staff were educated to wash hands with soap and water for all CDI patients. The enteric contact sign also instructs everyone entering and leaving the room to wash hands with soap and water.

Descriptive statistics were used to report the number of infections, number of patient days, hand disinfection compliance, length of stay, patient age, acuity (case-mix index), rate of CDI per 10 000 patient-days, rate of stage 2 pressure ulcers per 1000 patient-days and rate of deep pressure ulcers per 1000 patient-days. Hand disinfection compliance was based on using the appropriate solution for hand disinfection. While hand disinfectants are allowed for non-CDI patients, in order be compliant, use of soap and water was required for hand disinfection. The case-mix index was calculated by taking the total of all patient’s diagnosis-related group weights and dividing it by the total number of patients. The overall usage of antibiotics and the five most commonly prescribed antibiotics were collected monthly and standardized using defined daily doses (DDDs) per 1000 patient-days beginning April 2016 through September 2018 (post-BB).

A Poisson regression model was conducted to assess the relationship between the two periods of the study (pre-intervention and post-intervention). Additional endpoints included stage 2 pressure ulcers and deep pressure ulcers. The Poisson regression specification with a log link was used to compare the monthly counts of CDI and the secondary endpoints, adjusted for patient days. Two models were performed. The first model included only the BB variable. The second model included hand disinfection compliance, length of stay, case-mix index, and patient age.

All data analyses were performed using SPSS 24.0 (IBM, Armonk, NY). Graphics were produced using MS Excel (Microsoft, Redmond, WA). The study was reviewed and approved by the Institutional Review Board of Xavier University in Cincinnati Ohio.

## RESULTS

**Figure 1:**
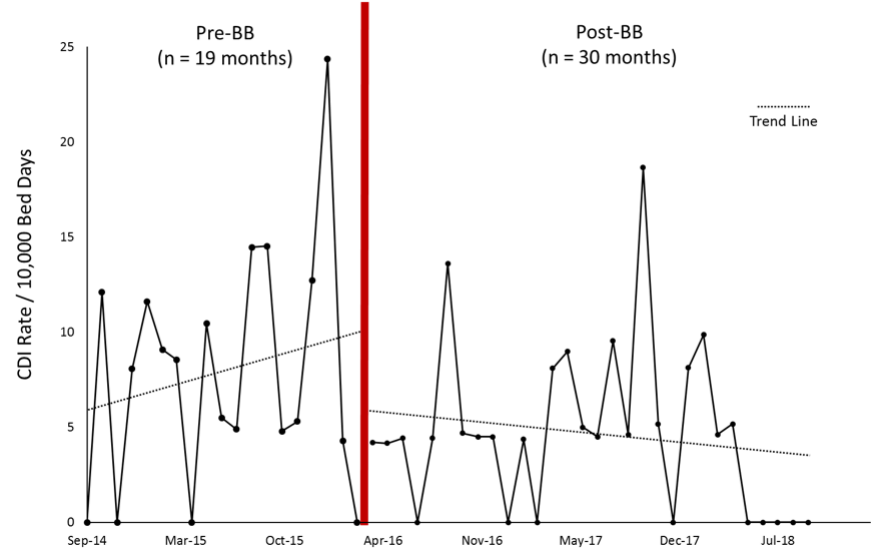
CDI rate per 10 000 patient bed days. The figure shows the rate of hospital onset *Clostridioides difficile* (HO-CDI) before and after the introduction the launderable bed barrier and antibiotic stewardship program.

There were 34 HO-CDIs and 42 672 patient days in the 19-month pre-intervention period and 31 HO-CDIs and 65 882 patient days in the 30-month post-intervention period (Figure 1). The corresponding average monthly rate (per 10 000 patient-days) was 7.94 (SD 6.30) and 4.71 (SD 4.42) during the pre- and post-periods (*p* 0.062). The mean age in the pre-intervention period was 58 (SD 1.90) years, and in the post-intervention period, it was 58 (SD 1.20) years (*p* 0.927). The mean hand disinfection compliance rate was 86% pre-intervention (IQR range, 64%–98%) and 87% post-intervention (IQR range, 75%–95%; *p* 0.463). Descriptive statistics for hand disinfection rates, acuity, pressure ulcers, and length of stay for the pre- and post-intervention periods are reported in Table 1.

During the study period, there was a 37% decline (*p* <0.0001) in the use of all antibiotics (957.4 to 600.8 DDD per 1000 patient days). Of the five most commonly prescribed antibiotics, ceftaroline had the largest percentage decline of 95% (7.50 to 0.40 DDD per 1000 patient days; *p* <0.0089) and daptomycin had the lowest percentage decline of 11% (16.50 to 14.70 DDD per 1000 patient days; *p* <0.2716) (Figure 2a and 2b). Detailed data was available for the following antibiotics/antibiotic classes: Vancomycin, Quinilones, Carbapenems, Ceftaroline, and Daptomycine (Table 3).

Poisson regression results indicated that the use of a bed barrier and antibiotic stewardship was associated with a statistically significant risk reduction of 59.1% (95% CI 36%–96%, *p* 0.034) in the occurrence of HO-CDI (Table 2). In the saturated model, which included the rate of hand disinfection compliance, length of stay, and acuity, the bed barrier and antibiotic stewardship program was associated with a 59.2% (95% CI 36–99%, *p* 0.033) reduction in the rate of HO-CDI. The differences in the rate of hand disinfection, length of stay, and acuity were not statistically significant in the saturated model.

Although the BB is made of the same breathable fabric as the mattress cover, its use does add a layer of material onto the mattress. Therefore, we tracked pressure ulcers (PU) during both phases of the study. Stage 2 PUs and deep PUs were tracked during the pre- and post-periods of the study. Data was missing for the first 4 months of the pre-intervention period. The results of the stage 2 PU secondary analysis did not find a statistically significant increase in PUs in the reduced (*p* 0.135) or saturated (*p* 0.226). Likewise, the results of the deep PU secondary analysis did not find a statistically significant increase in the reduced (*p*=0.739) or saturated (*p* 0.876) model specification (Table 2).

## DISCUSSION

In an acute care hospital, we found that the concurrent use of a BB and ASP resulted in a 59% reduction in HO-CDI. Our study suggested substantial reductions of HO-CDIs can still be achieved above and beyond terminal cleaning with the introduction of a BB and ASP. Recent national attention to HO-CDIs has resulted in a plateau in the rate of infections for the United States. However, the clinical and economic burden is still substantial with an estimated cost to hospitals of nearly $5 billion per year.^3^,^6^ The economic cost combined with the Center for Medicare and Medicaid Services (CMS) reimbursement penalties for healthcare associated infections, including HO-CDI, requires hospitals to explore opportunities to further reduce their rates.

Previous studies have shown that ASPs can decrease HO-CDIs; however, it is unlikely to have accounted for the entire 59% decrease in HO-CDIs.^6^ A 2015 study using the BB, without any changes in antibiotic stewardship, showed that it was associated with a 50% decrease in HO-CDIs in two LTACHs.^22^ Unfortunately, our study was not designed in a manner to isolate the individual effects of a launderable BB and ASP in the reduction of HO-CDI.

The BB provided a mattress surface free of pathogenic bacteria and *C. difficile* spores for each patient. The covers were cleaned using a validated laundry process, which resulted in greater than a log 6 reduction (99.9999%) in pathogenic bacteria and *C. difficile* spores.^26^ A recent report that showed that use of commercial laundry failed to remove *C. difficile* spores from linens.^27^ The success of the laundry process in disinfecting the BB was likely due to it being fabric coated with polyurethane, which allowed the laundry process to successfully remove the *C. difficile* spores. Terminal cleaning of the hospital room, including the bed and mattress, has been performed using a number of different chemicals and ultraviolet light (UV light). These chemicals included quaternary ammonia compounds (Quats), phenolic cleaners, hydrogen peroxide/peroxyacetic acid (peracetic acid), and sodium hypochlorite (bleach). Unfortunately, these chemicals often failed to achieve the appropriate level of disinfection in current practice. Quats only achieve a log 1 (90%) reduction of pathogenic bacteria.^13^,^28^,^29^ Peracetic acid has been shown to get a log 2 (99%) reduction in pathogenic bacteria, but it was only shown to achieve only a log 1 (90%) reduction of *C. difficile*.^30^,^31^ Also, peracetic acid use failed to decrease HAIs in a single hospital study.^32^ Bleach is frequently used by hospitals for rooms known or suspected to be contaminated with *C. difficile*. In laboratory studies, high concentrations (5000 ppm) of bleach have been shown to effectively lower bacterial and spore counts by up to log 5 (99.999%) reduction. However, many hospital surfaces are still contaminated with *C. difficile* after application of bleach (less than a log 1 reduction).^33^,^34^ UV light fails to reduce counts of *C. difficile* spores, and failed to decrease infections with *C. difficile* when studied.^34^

The other issue for all of the disinfectants being used currently to clean hospital rooms, including the mattress, is the fact that these disinfectants are only approved for use on hard non-porous surfaces. Hospital mattresses were originally made of non-porous vinyl. Due to concerns over skin breakdown and pressure ulcers, mattress covers are now made of soft, porous material. They are commonly made of porous materials including nylon covered by polyurethane or woven nylon backed with polyurethane. Major bed manufacturers have instructions for use (IFUs) of their product, and the most recent IFUs recommend using a multistep process that includes precleaning, cleaning, rinsing, disinfecting, rinsing the disinfectant, and inspecting the mattress for damage with a separate set of disinfectants than the ones used for hard surfaces.^35^,^36^ The CMS requires hospitals to follow the manufacturer’s IFU to ensure proper reprocessing of hospital beds and mattresses.

The bed manufacturers and the FDA recommend routine inspection of the mattress for damage.^16^,^35^,^36^ Two large studies have shown that between 25% and 33% of mattresses have damage and up to 4% have fluid inside of them.^14^,^15^ Inspection of the mattress requires it to be unzipped to evaluate for damage and fluid inside. The BB used in this study not only protects the bed frame and mattress from damage but also was inspected for damage using a light table after each laundering. The BB used in this study was laundered using detergent, bleach, hot water (71ºC) and multiple rinses.

## LIMITATIONS

The results of this study must be interpreted in light of several limitations. The study was performed at one acute care hospital. The study was a pre-post study design, which is susceptible to confounding. Potential confounders were changes in antibiotic stewardship, change in diagnostic testing, decreased use of proton-pump inhibitors, and improved environmental cleaning. Though, environmental cleaning companies remained the same during all periods of the study. The number of antibiotic days, days in which patient was exposed to any antibiotic, was not measured during the study period, and the use of proton-pump inhibitors was not monitored during the study. Finally, the study was not designed to ascertain the individual effects of a launderable BB and ASP.

There was a change in testing methodology for CDI during the study. The hospital moved from doing a NAAT test to using the ELISA test for toxin A and B only for cases starting on day 4 of hospitalization or later. Some of the decrease seen in rates of CDI at the hospital may be attributable to the use of a more specific test. However, the new ELISA tests for toxin A and B have much higher sensitivities, and their use should have only accounted for a small decrease in the rate of CDIs.^37^ Though promising, the results of this study should be viewed as a feasibility study due to the limitations noted.

## CONCLUSIONS

The use of a launderable BB and ASP was associated with a 59% decrease in HO-CDIs at an acute care hospital. Hospitals should consider using a launderable BB and aggressive antibiotic stewardship in order decrease hospital-onset *Clostridioides difficile* infections.

## Figures and Tables

**Figure 2a:**
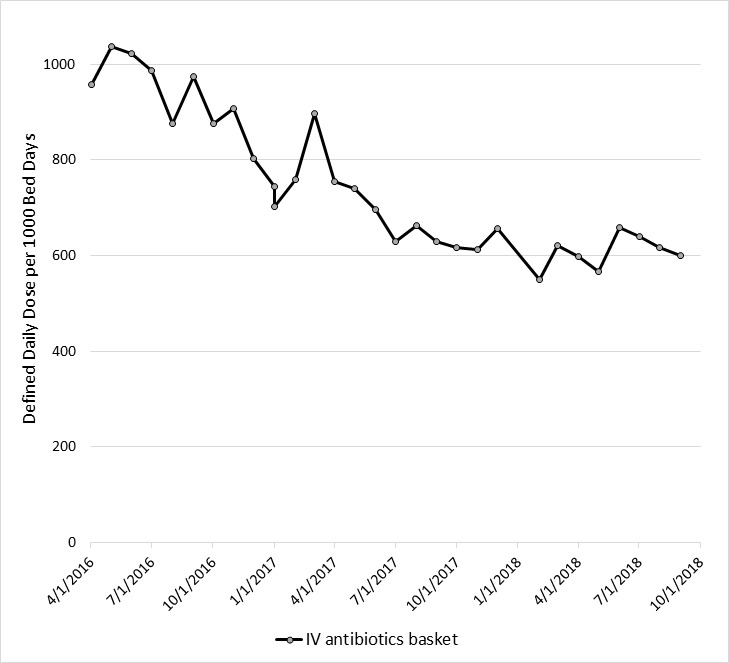
Overall Antibiotic Usage During the Post-BB and ASP period

**Figure 2b:**
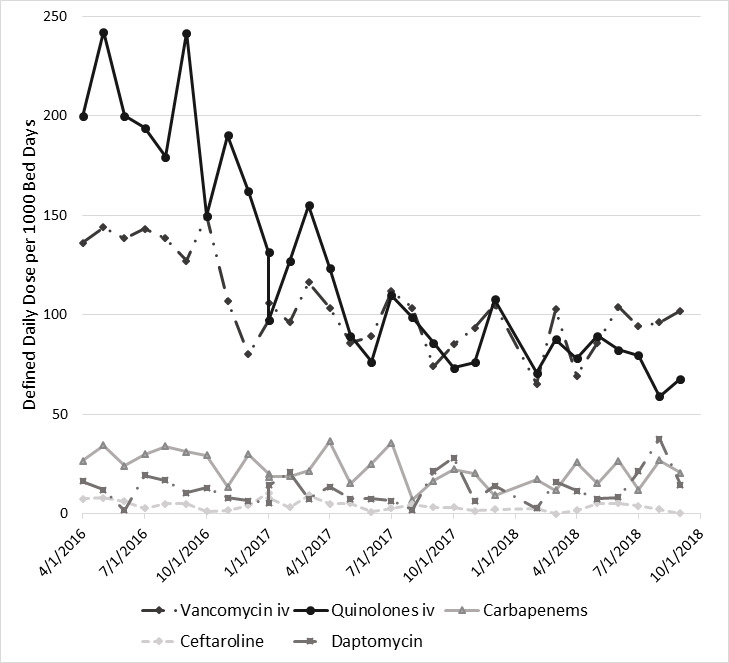
Antibiotic Usage During the Post-BB and ASP Period for the Five Most Commonly Prescribed Antibiotics

**Table 1:** Descriptive statistics for HO-CDI, Hand disinfection, Acuity, Age, and Length of Stay

	Pre-Bed Barrier (19 months)				Post-Bed Barrier (30 months)				
	Mean	SD	Median	IQR	Mean	SD	Median	IQR	*p* value
CDI Rate / 10 000 patient days	7.94	6.30	8.07	4.29–12.11	4.71	4.42	4.50	0.00–5.91	0.062
Stage 2 PU Rate / 1000 patient days*	1.35	2.42	0.00	0.00–3.87	3.04	4.19	0.00	0.00–4.53	0.094
Deep PU Rate / 1000 patient days*	3.96	3.81	4.24	0.00–7.31	4.22	4.97	4.49	0.00–5.90	0.851
Hand Disinfection Compliance, %	85.89	8.46	87.00	81.00–91.00	87.47	4.52	88.00	84.00–91.00	0.463
Case Mix Index	1.49	0.08	1.51	1.43–1.54	1.48	0.04	1.47	1.46–1.50	0.654
Length of Stay, days	4.40	0.27	4.44	4.15–4.62	4.50	0.33	4.48	4.24–4.69	0.226
Average Age of Patients	58.24	1.90	58.80	56.40–59.70	58.29	1.18	58.40	57.50–58.93	0.927

SD: Standard Deviation; IQR: Inter-quartile range

Stage 2 PU Rate / 1000 patient days & Deep PU Rate / 1000 patient days: We assessed the occurrence of stage 2 pressure ulcers and deep pressure ulcers.

**Table 2:** HO-CDI Regression Analysis

Parameter	Coefficient	SEM	Lower	Upper	Wald X2	p value	Exp(B)	Lower	Upper
**Model 1**									
Intercept	−7.135	0.171	−7.471	−6.799	1730.849	<0.001	0.001	0.001	0.001
Bed Barrier	−0.527	0.248	−1.013	−0.040	4.498	0.034	0.591	0.363	0.961
**Model 2**									
Intercept	−3.962	6.267	−16.245	8.332	0.400	0.527	0.190	0.000	4115.27
Bed Barrier	−0.563	0.264	−1.079	−0.046	4.558	0.003	0.570	0.340	0.955

Model 1 only included the bed barrier. Model 2 included hand disinfection compliance, length of stay, case-mix index, and patient age. None of which were found to be statistically significant with a p value less than 0.05.

**Table 3:** Antibiotic Usage Pre-/Post-Bed Barrier, Defined Daily Dose per 1000 inpatient days

	Pre-Bed Barrier	Post-Bed Barrier	Difference	% Difference	p value
Vancomycin	136.3	102.1	−34.2	−25.1%	<0.0001
Quinolones	199.8	67.8	−132	−66.1%	<0.0001
Carbapenems	26.7	20.7	−6.0	−22.5%	0.0131
Ceftaroline	7.5	0.4	−7.1	−94.7%	0.0090
Daptomycin	16.5	14.7	−1.8	−10.9%	0.2951
IV antibiotics (n=30) includes above antibiotics*	957.4	600.8	−356.6	−37.2%	<0.0001

* The basket included 30 different IV antibiotics and 5 individual antibiotics were tracked separately.
