# Author Correction: Use of a Launderable Bed Barrier and Antibiotic Stewardship to Decrease Hospital Onset *Clostridioides difficile* Infections in an Acute Care Hospital: A Retrospective Pre/Post Case Study

**DOI:** 10.36469/jheor.2021.23476

**Published:** 2021-05-18

**Authors:** Edmond A. Hooker, Peter J. Mallow, Christine McKinney, Martin L. Gnoni, Francisco Fernandez Gonzales

**Affiliations:** 1 Xavier University, United States of America; University of Cincinnati, United States of America; 2 Xavier University, United States of America; 3 Our Lady of Bellefonte, United States of America

**Keywords:** antibiotic stewardship program, bed barrier, bacterial infection, clostridioides difficile

Correction to: https://doi.org/10.36469/001c.11149

Published online: Dec 12, 2019

We noticed an issue in the reporting of the Results in this Article. Specifically, the Odds Ratios (ORs) were reported as a percent decrease in hospital-onset *Clostridioides difficile* infections (HO-CDIs) as a result of adopting a launderable bed barrier and an antibiotic stewardship program throughout the Article. We have updated the primary results replacing a 59% decrease with a 41% decrease and restating the (OR=0.59; 95% confidence interval [CI] 0.36-0.96; *P*=0.034) throughout the appropriate sections of the manuscript. Similarly, Model 2 was misreported in the Results section and has been updated to reflect the 43% decrease in HO-CDI (OR=0.57; 95% CI 0.34-0.96; *P* 0.003). These corrections do not change the primary finding that the mattress barrier and an antibiotic stewardship program were associated with a significant reduction in HO-CDI. The original Article has been updated online.

